# Characterising phase variations in MALDI-TOF data and correcting them by peak alignment

**Published:** 2007-02-23

**Authors:** Simon M Lin, Richard P Haney, Michael J Campa, Michael C Fitzgerald, Edward F Patz

**Affiliations:** a Duke Comprehensive Cancer Center; b Department of Radiology; c Department of Chemistry and; d Department of Pathology; e Department of Pharmacology and Cancer Biology, Duke University and Medical Center, Durham, NC 27710, USA; f Robert H. Lurie Comprehensive Cancer Center, Northwestern University, Chicago, IL, USA

**Keywords:** variation, amplitude, phase, MALDI-TOF, peak alignment

## Abstract

The use of MALDI-TOF mass spectrometry as a means of analyzing the proteome has been evaluated extensively in recent years. One of the limitations of this technique that has impeded the development of robust data analysis algorithms is the variability in the location of protein ion signals along the x-axis. We studied technical variations of MALDI-TOF measurements in the context of proteomics profiling. By acquiring a benchmark data set with five replicates, we estimated 76% to 85% of the total variance is due to phase variation. We devised a lobster plot, so named because of the resemblance to a lobster claw, to help detect the phase variation in replicates. We also investigated a peak alignment algorithm to remove the phase variation. This operation is analogous to the normalization step in microarray data analysis. Only after this critical step can features of biological interest be clearly revealed. With the help of principal component analysis, we demonstrated that after peak alignment, the differences among replicates are reduced. We compared this approach to peak alignment with a model-based calibration approach in which there was known information about peaks in common among all spectra. Finally, we examined the potential value at each point in an analysis pipeline of having a set of methods available that includes parametric, semiparametric and nonparametric methods; among such methods are those that benefit from the use of prior information.

## Introduction

MALDI-TOF and SELDI-TOF have been used to profile the proteome for biomarker discovery and cancer diagnostics (Adam et al., 2002; Campa et al., 2003b; Li et al., 2002; Wulfkuhle et al., 2001). For a review, see (Wulfkuhle et al., 2003). Data analysis has been identified as a bottleneck for these proteomics studies (Campa et al., 2003a). In doing the data analysis, typical approaches involve a pipeline of approaches that start with denoising and background correction of the signals, include steps such as the handling the multiple isotopes, and conclude with high-level analyses such as biomarker discovery or survival analysis. One of the steps in such a pipeline involves handling the technical variations in MADLI-TOF and SELDI-TOF measurements that occur from run to run. It is that step of the mass spectrometry data analysis pipeline that we examine in detail in this paper.

In our MALDI-TOF experiment protocol, we typically collect two to ten replicate spectra from the same biological specimen by sampling different spots on the MALDI plate (Wang et al., 2003). Combining these replicates into a composite spectrum is the first step to reduce raw data and extract signals of biological interest.

Time-of-flight (TOF) data has both *amplitude* variation and *phase* variation: not only does the intensity at a certain peak vary, but also the location of the peak on the time-of-flight, or x-axis. Even though we do mass calibration every time we use the equipment, we still see phase (mass) variations from run-to-run due to technical instabilities in the apparatus. It was documented that the accuracy of the peak location in MALDI-TOF is within 0.15% to 0.3% of the mass (m/z) value (Campa et al., 2003b). This phase variation was one of the obstacles encountered during analysis of the lung cancer data set in the first annual Proteomics Data Mining Conference (Campa et al., 2003a).

The phase variation problem is not unique to MALDI-TOF data, but also exists in SELDI-TOF data. James Lyons-Weiler reported the phase variation problem in SELDI-TOF data sets, and referred to it as profile alignment or the mass calibration problem (Lyons-Weiler et al., 2003). In a SELDI-TOF study of ovarian cancer, Thomas Conrads and colleagues pointed out that in order to generate better discriminations of biological interest, the peak alignment problem should be solved first (Conrads et al., 2004). The peak alignment problem itself also occurs in other mass spectrometry settings, such as in the alignment of LC retention times in LC-MS analyses. Aligning curves through transformation of time, as in the time-of-flight axis in MALDI-TOF, is generally called *time warping* in the engineering literature (Sakoe & Chiba, 1978) and *curve registration* in the statistical literature (Ramsay & Li, 1998).

When analyzing such problems, there may be value in having a set of methods available at each point in the analysis. In some cases, for example, we may at first have no prior information on known calibrants. For example, in analyzing MALDI-TOF data we may have prior information as to the exact m/z values of known calibrants or contaminants. In that circumstance, it is valuable to have a method that can make use of the prior information on those calibrants or contaminants. On the other hand, in other analyses, such as those used for aligning LC retention times for current LC equipment, it is possible that no accurate method using prior information involving the biophysics of the system will ever be available. This suggests the utility of developing an overall family of methods, some of which can benefit from specific prior information, and some of which do not.

## Related work

Previous attempts to solve the phase variation problem in MALDI-TOF have included binning adjacent peaks by the 0.1% rule (Hilario et al., 2003; Wang et al., 2003) and grouping the peaks by clustering algorithms (Lin et al., 2003; Slotta et al., 2003). A prerequisite of all of these aforementioned methods is that the peaks must be identified first. Here we present an approach to improving the peak identification process that is based on a new method to align the replicates first. The subsequent peak detection process can be improved once the aligned spectra are averaged.

In this paper we study the overall variations in MALDI-TOF data, to detect phase variations we look at a lobster plot (named because of the resemblance of a lobster claw), and we examine an established curve registration algorithm to correct the phase variations. In an on-line appendix at http://dbsr.duke.edu/pub/alignPeaks/, we note that one perspective on analyzing spectra is that work in this area represents a problem of function estimation. In that context, there are a variety of measures (e.g., Hellinger distances and symmetric Kullback-Liebler divergence) to determine the overall quality of a given function estimation process. In that appendix, we also see if lobsterplots could be useful, heuristic devices that can help us visually represent Hellinger and other measures of model fitness.

## MALDI-TOF data set and data preprocessing

We acquired a data set with five replicates to investigate the technical variability of MALDI-TOF data. Each replicate was derived from the same lysate of normal lung tissue. Details of the experimental preparations have been previously described (Campa et al., 2003b). We believe this data set, consisting of spectra of actual biological samples, to be preferable to a fully simulated one to study technical variations.

To illustrate the principles of our alignment algorithm and to reduce computational loads for the initial investigation, we picked two segments (14.861K to 15.265K, and 15.717K to 16.132K in m/z) of the spectra in this study. Briefly, we artificially segmented two distinct mass ranges from the same biological sample and treated them as coming from two different biological samples in the same mass range. This manipulation artificially creates two biological samples but preserves the characteristics of the raw measurements from the MALDI-TOF instrument. In the following discussion, we refer to these simulated samples as biological sample A and B. For each biological sample, we have five replicates, namely, A1-A5 and B1-B5.

The raw intensities were square-root transformed, and then subjected to a baseline correction procedure by subtracting the 25^th^ percentile of the intensity values, followed by a rescale procedure to project the data to the (0, 1) range. A plot of the spectra after preprocessing can be found in Figure 3a and 3b. TOF data were usually collected by binning time into short intervals and then counting the intensities during each interval. To plot the spectrum, we simply plot the intensity against the bin number, instead of using the more common m/z value. The m/z value is related to the bin number by a monotonic function.

## Technical variations due to phase shift

**Figure 1:**
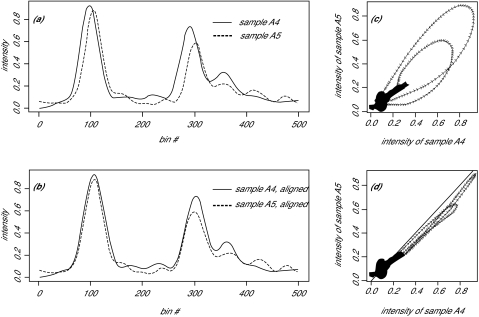
MALDI-TOF spectrum of a pair of replicates A4 and A5. Shown are spectra before (a) and after (b) alignment. The corresponding lobster plots are shown in (c) and (d).

To illustrate technical variations due to phase shift, we plotted the spectra of a pair of replicates, sample A4 and A5, both of which are from the same biological sample A (Figure 1a). Under ideal situations with no technical variation, these two curves should overlap. The real data demonstrates some inconsistency. We can measure inconsistency among the samples using a “distance” measure of some kind (such as a Kullback-Liebler divergence measure or Hellinger distance); we make direct use of one of those measures below. However, in addition to having quantitative metrics of the inconsistency, it is helpful to have visual representations of the differences. We therefore start by plotting the intensities at each bin using a scatter plot to visually examine the curves.

In particular, we wanted to determine whether the inconstancy was due to amplitude differences or phase variations. Thus, we also devised a “lobster plot” to connect the consecutive points (in time-domain) in the regular scatter plot with an arrow. In this plot, phase shifts between the pair of measurements can be detected by loops formed in the shape of a lobster claw (Figure 1b). The direction of the phase-shift can be detected by the clockwise or anticlockwise loop of the lobster claw.

Phase variations cause two potential problems in proteomic profiling. First, we cannot simply average the replicates to generate a composite spectrum that is representative of the biological sample. A simple average can result in a distorted estimate not resembling any sample curves (Ramsay & Li, 1998). For example, the shape of some peaks may be flattened due to averaging over out-of-phase alignments. This problem is especially a nuisance in many analytical chemistry data, where the shape and area of the peak can convey chemical information.

Second, the phase variation in replicates makes computerized pattern analysis more difficult. Human eyes are quite adept at recognizing shifted signals, but multivariate statistical algorithms such as principal component analysis (PCA) cannot distinguish the phase variation from real biological differences. We will discuss an algorithm to remove the phase variation and demonstrate its effect on reducing within-sample variation in the following sections.

## Aligning shifted peaks in replicates

Phase variation problems have previously been studied in many other types of scientific measurements, such as speech recognition (Sakoe & Chiba, 1978), chromatograms (Nielsen et al., 1998), and NMR spectra (Brown & Stoyanova, 1996). Just as in MALDI-TOF data, the peak shifts are not uniform throughout the whole spectrum: some peaks can be perfectly aligned, while other peaks either shift ahead or behind. Thus, we need an algorithm to align the local features in the spectra. We especially prefer an algorithm that does not require the location of the peak to be specified first.

Solutions to this problem include parametric, semiparametric and nonparametric methods. One example of a nonparametric approach involves the use of dynamic programming to align the observations. While Sauve and Speed (2004) give encouraging results, one major problem of is the introduction of sudden jumps and plateaus, which has undesired effects on analytical chemistry signals (Pravdova et al., 2002 and Eilers, 2004).

Other approaches involve model-based calibration involving known locations of peaks occurring in common among many spectra. In this approach, the sets of peaks in common are found with the help of one or more automated peak finding algorithms. At that point a calibration (or recalibration) model aligns the known targets, typically using either a global linear or quadratic model, or, if a global calibration curve cannot be established, a more local, piecewise linear model. Wool and Smilansky (2004) explore different aspects of this approach in detail. We also examine one particular version of this approach below.

Another approach involves the use of splines and functional data analysis (FDA) together with a curve registration process (Eilers, 2004; Ramsay & Li, 1998). In that context, suppose we have a spectrum *x(t)* to be aligned to the template spectrum *x_0_(t).* We want to find a flexible yet monotonic increasing function *h(t)* such that *x[h(t)]* is as close as possible to *x_0_(t).* Function *h(t)* is called the warping function, since the transformation of time corrects the phase shift problem. The fitness of *h(t)* can be evaluated with the familiar least square criterion,

$$Fitness(h)=\int {\left\{x_{i}[h(t)]-x_{0}(t)\right\}}^{2}dt$$

As implied above, we can use other measures (often quadratic distance measures) to assess the fitness.

In this context, we made use of an R program library by Ramsey that models *h(t)* by B-splines (Ramsay & Li, 1998). We applied this method to the pair of replicates in Figure 1a. The spectra of sample A4 and A5 after alignment are shown in Figure 1c.

From their corresponding lobster plot (Figure 1d), we can see the phase-shift being corrected by the closing of the lobster claws.

## Extending the method to multiple replicates

**Figure 2:**
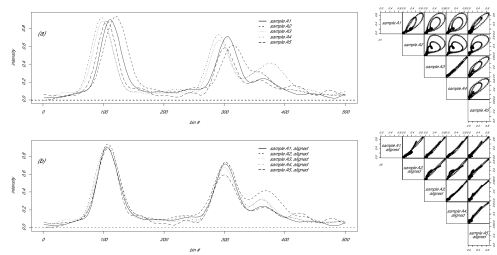
Five replicates from sample A. Shown are the spectrum (a and b) and corresponding lobster plot (c and d) before (a and c) and after (b and d) time warping.

So far we have discussed the phase variation problem and its solution in a pair of replicates. This methodology can easily be extended to handle multiple replicates. Similar to the extension of microarray normalization beyond pair wise, we can devise three solutions. As a first solution, we can designate one spectrum as the template, and then align the other spectra to this template. As a variation of the first method, we can derive the second method by using the average of all spectra as the template. We used this second solution in this paper. Furthermore, we can implement a more computationally intensive third solution by alternating the averaging and aligning steps until some convergence criterion is met. The lobster plot can also be extended to handle multiple spectra by a pair wise strategy similar to pair wise scatter plots (Figure 2b and Figure 2d).

As discussed before, technical variations of MALDI-TOF data can be partitioned into amplitude variation and phase variation. To characterize the significance of phase variation in the five replicates, we calculated the total variance before and after time warping for sample A and B, respectively (Figure 2 and Table 1). After curve registration, we can eliminate 84% and 76% of total variance for sample A and B, respectively.

## PCA analysis of replicates from sample A and B

**Figure 3:**
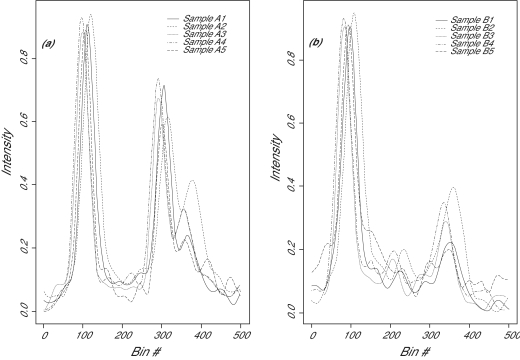

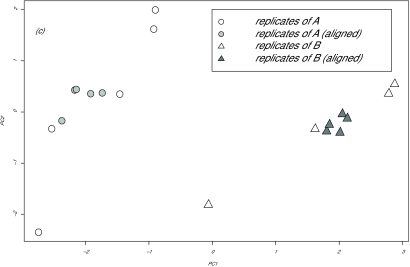
Spectrum from two different biological samples A and B. (a) Replicates of sample A1 to A5. (b) Replicates of sample B1 to B5. (c): PCA plot of all spectra before (open symbols) and after (filled symbols) peak alignment. Note that one of the open triangles is hidden behind the filled triangles.

The relationships among spectra can be visualized by an unsupervised pattern recognition algorithm PCA (Lee et al., 2003). One reason to use PCA is that it projects the data points in a high-dimensional space into a 2-dimension space that works better for visualization. PCA works by combining many original features into a very few principal components (Jolliffe, 1986). In the PCA plot (figure 3C), each spectrum is represented as a single point. The distance between these points is a function of the similarity of the spectra: the closer the points, the more similar the spectra. As seen in Figure 3c, the spread of the replicates are tightened after the peak alignment (filled symbols).

## Comparison of this peak alignment method with model-based calibration

We consider the above approach to be one of a family of methods that also includes model-based calibration involving known targets. In that approach, unlike the approach above, an automated peak finding algorithm is used that finds peaks in common within sets of spectra. While there are various nuances when comparing the various approaches, the results can be summarized as follows:

- Consistent with the authors’ prior experiences with Bayesian models, approaches involving the use of an automated peak finding algorithm that followed by model-based calibration can be more effective than our FDA-based method as described above. This is particularly the case if the phase variation (that is, the way by which spectra are “warped”) involves a constant shift, or is a linear function.
- The FDA-based method at hand works well when there is no prior information involving peaks. This situation arises during some analysis pipelines, in particular with MALDI-TOF data. In other words, the method is of use (a) during completely automated analyses, (b) when there are no guaranteed peaks in common among the spectra being analyzed, or (c) when done during an initial alignment step that occurs prior to discovery of peaks in common among all the spectra.
- When using simulated data, if the function defining the phase variation (i.e. the warping function) is not constant, linear, or quadratic, then the method at hand is more effective than the use of model-based calibration. Such a situation arises in some cases when aligning the LC retention times of mass spectrometry peaks.

While FDA-based approach also innately smoothes data using B-splines, in order for it to work well on datasets other than MALDI-TOF datasets, such as those from Fourier Transform Ion Cyclotron Resonance (FTICR) devices, we have found it necessary to apply additional smoothing and denoising techniques.

**Figure 4:**
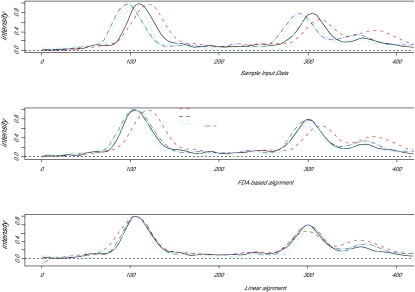
Comparison of initial data, FDA-aligned data, and alignment using prior information.

We illustrate the first result in Figure 4. The first plot shows unaligned peaks, with the second showing peaks aligned using the approach outlined. The third shows peaks aligned with the help of automated peak finding and then “forced” into alignment using a piecewise linear (linear spline) model. This alignment is in spite of some data for the A5 curve that suggests this replicate is in fact quite different in nature. In the “forced alignment”, modest evidence to the contrary is not allowed to trump the initial prior information guaranteed by the researcher to be correct.

In this scenario, if it is known for sure that main peaks are in common, then the third approach is simpler and more effective than the approach discussed above.

## Discussion and Conclusions

We characterized the phase variation in replicates of MALDI-TOF data. These phase variations account for 76% to 85% of the total variation in the replicates. We devised a lobster plot to detect the phase variation, and a peak alignment algorithm to remove the phase difference. We then compared this peak algorithm to an approach of model-based calibration. We are in the process of adding in an additional, completely nonparametric approach of dynamic time warping or DTW. We have concluded that all have a role, as part of an overall set of methods that include parametric, semiparametric and nonparametric methods, some of which can benefit from the availability of prior information. In this context, we plan to add additional methods to the existing two approaches coded thus far.

Overall, and in particular in comparison to the use of dynamic time warping, we would characterize the FDA-based method we used as semiparametric. However, we would caution that it is not the only one in its category; other methods using splines are apt to contain relatively more (or fewer) parametric aspects. One benefit of the specific choice we used is that can also be coupled to doing functional analysis of variance, or FANOVA.

We noted in the results section that lack of smoothness in the underlying data (or a situation in which smoothing is undesirable) also affects the results when using methods that, as in the case of the FDA method, can make use of information involving the derivatives of the curve. We also compared the method to a model-based calibration approach that makes use of prior information of internal and external calibrants. As might be expected, such an approach using known lists of common peaks can be more effective than the approach above. However, not permitting the data itself to ever outweigh that particular prior information also carries own risks.

Overall, we have concluded that the approach above, based upon the use of functional data analysis (FDA) represents one useful method that is part of an overall mix of approaches that includes parametric, semiparametric and nonparametric approaches to the task of peak alignment and calibration.

## Figures and Tables

**Table 1:** Percentage of variation explained by time warping

	Variance due to phase	/total variance	= % of variance due to phase
**Sample A**	3.37	3.97	84.98%
**Sample B**	2.52	3.31	76.28%
